# How to successfully implement population health management: a scoping review

**DOI:** 10.1186/s12913-023-09915-5

**Published:** 2023-08-25

**Authors:** A. F. T. M. van Ede, R. N. Minderhout, K. V. Stein, M. A. Bruijnzeels

**Affiliations:** Department of Public Health and Primary Care/ Health Campus The Hague, Leiden University Medical Centre, The Hague, The Netherlands

**Keywords:** Population Health Management, Transformation, Implementation

## Abstract

**Background:**

Despite international examples, it is unclear for multisector initiatives which want to sustainably improve the health of a population how to implement Population Health Management (PHM) and where to start. Hence, the main purpose of this research is to explore current literature about the implementation of PHM and organising existing knowledge to better understand what needs to happen on which level to achieve which outcome.

**Methods:**

A scoping review was performed within scientific literature. The data was structured using Context-Mechanism-Outcome, the Rainbow model of integrated care and six elements of PHM as theoretical concepts.

**Results:**

The literature search generated 531 articles, of which 11 were included. Structuring the data according to these three concepts provided a framework that shows the skewed distribution of items that influence the implementation of PHM. It highlights that there is a clear focus on normative integration on the organisational level in ‘accountable regional organisation’. There is less focus on the normative integration of ‘cross domain business model’, ‘integrated data infrastructure’, and ‘population health data analytics’, and overall the perspective of citizen and professionals, indicating possible gaps of consideration.

**Conclusions:**

A first step is taken towards a practical guide to implement PHM by illustrating the depth of the complexity and showing the partial interrelatedness of the items. Comparing the results with existing literature, the analysis showed certain gaps that are not addressed in practice, but should be according to other frameworks. If initiators follow the current path in literature, they may be missing out on some important components to achieve proper implementation of PHM.

**Supplementary Information:**

The online version contains supplementary material available at 10.1186/s12913-023-09915-5.

## Background

The Netherlands, like many countries, faces challenges in healthcare provision to its population and in the healthcare system. The healthcare system aims to provide care for all inhabitants, assure a high quality of care, keep care affordable and ensure people remain in good health and therefore prevent the need for care. Factors like increasing healthcare expenditures, an ageing population with increasing multimorbidity, and the burden of a shrinking workforce make achieving these goals challenging [1]. As a result, the long-term sustainability of the system is under pressure. To summarise what the main aims of a health system should be, Berwick introduced the Triple Aim approach, which later evolved into the Quadruple Aim [2, 3]. This is described as a simultaneous pursuit of improving the experience of care, improving the health of populations, reducing per capita costs of healthcare, and improving the work life of healthcare professionals [4]. In recent years, Population Health Management (PHM) has emerged as an approach to achieve the Quadruple Aim and foster the necessary systemwide changes [5]. PHM can be summarized as the proactive management of a total population at risk through a variety of individual, organisational and cultural interventions to improve health and financial outcomes [6, 7]. Successful implementation of PHM is seen in examples all around the world, like the Accountable Care Organisations in the US and regional initiatives like Greater Manchester in the UK and Gesundes Kinzigtal in Germany [8, 9]. In the Netherlands, the non-governmental organisation HealthKIC has started to implement PHM using the so-called ‘PLOTmodel’ (Kavelmodel in Dutch), referring to regions as plots. Despite many international examples and case studies into the “what” of PHM implementation, the “how” remains elusive. While there are many frameworks defining the different items of health system transformation, so far no guide exists on how to prioritise the many different changes necessary and indeed where to start. Hence, the main purpose of this research is to explore current literature about the implementation of PHM by organising this existing knowledge to better understand what needs to happen on which level to achieve which outcome.

In current literature, more and more studies describe the implementation of whole system transformation initiatives [10–13]. However, different perspectives and methods are used to order the themes that influence implementation. For instance, Suter et. al. described ten key principles for health system integration: comprehensive services across the care continuum, patient focus, geographic coverage and rostering, standardized care delivery through interprofessional teams, performance management, information systems, organisational culture and leadership, physician integration, governance structure, and financial management [11]. The World Health Organization (WHO), in their framework on integrated people-centred health services, proposed five strategies that should be adopted: empowering and engaging people and communities, strengthening governance and accountability, reorienting the model of care, coordinating services within and across sectors and creating an enabling environment [12]. And in addition, the latest scoping review on this topic from Steenkamer et. al. described eight components of reorganizing and integrating the health system: social forces, resources, finance, relations, regulations, market, leadership, and accountability [13]. However, this ambiguous classification of themes that influence the implementation of a new health system approach does not support the implementation of PHM in practice as these are mostly lists of activities of what to do, with no specification as to how and when to address them, and on what level within the system.

Acknowledging health systems as complex adaptive systems, the difficulty of providing a predictable blueprint or framework becomes apparent. Complex adaptive systems are characterized by a high degree of interrelatedness, the decomposability of components, and the occurrence of unintended outcomes [14]. Supporting people in the transformation thereof thus requires more than a themed list of items that are important. Using other frameworks to break down the complexity of practice might add to our understanding. One of the frameworks that could contribute is the Context, Mechanism, and Outcome (CMO) framework [13]. The CMO is often used in process evaluations of complex interventions to describe what works for whom under what circumstances [15]. This distinction makes it easier to translate lessons learned into a different context [16]. Another framework that is often used in integrated care research is the Rainbow Model of Integrated Care (RMIC). According to the RMIC, there are four different levels of integrated care: clinical, professional, organisational, and system integration and two types of integration: normative and functional [17]. Using this framework helps assessing which actions need to take place at the different levels of the health system. Furthermore, the PLOTmodel uses six elements of PHM to guide its strategy: accountable regional organisation, cross domain business model, integrated data infrastructure, co-designing workforce and community, population health data analysis, and emergent implementation strategy [18].

To conclude, it remains unclear how new initiators that want to start with the implementation of PHM, such as the PLOTmodel, should be supported with knowledge about what to do when [10, 19]. Unravelling the complexity of implementing PHM by ordering the numerous items into different theoretical concepts may contribute to close this knowledge gap and may simultaneously increase the practical applicability of this knowledge for new and existing PHM initiators in their whole system transformation challenge. For this reason, this research attempts to answer the following questions: *What guidance can literature give us on the implementation of PHM? Does using the CMO, the RMIC, and the six elements of PHM help our understanding about the implementation of PHM in practice?*

## Methods

### Study design

To address the research questions, a scoping review was performed using the six-stage methodological framework from Arksey and O’Malley [20]. Since the focus of the research is on the current state of evidence in the field, the scoping review was considered most fitting. The use of this type of review allowed for a quick examination of the current scientific literature, without comprehensively having to evaluate the quality of the included articles [21].

### Inclusion criteria

To search for relevant articles, a search string was built together with a librarian. The search string contained the following keywords: population health management, integrated care, accountable healthcare, healthcare system, transformation. Specific diseases that were frequently found in the search results were excluded to improve the search string (see additional file 1). The electronic databases Embase, PubMed, and Academic Search Premier were searched for papers published between January 2016 and January 2021. The focus on the literature of the last 5 years was deemed appropriate as PHM is quite a recent development in health system transformation and the field is very dynamic and fast-paced.

The reference details and abstracts retrieved from the literature search were downloaded to Endnote X9, a bibliographic management software program. The articles were screened by title and abstract independently by two researchers (AE and NM) to identify their relevance. Articles were considered relevant if they described at least two themes that influence a transformation in healthcare within a multisector initiative, with at least three disciplines involved. When considered relevant by both researchers, the full text of the paper was retrieved and screened independently by two researchers (AE and NM). Within the selection process, relevance was discussed after screening the first set of articles on title and abstract, before retrieving full articles, and after full text screening to reach consensus between the researchers. If no agreement was reached, a third researcher was consulted. As part of the consultation phase, a scientific expert was consulted whether key studies were missed out on.

### Data analysis

Data analysis was done based on three frameworks:

- CMO was adopted to consider what works for whom under what circumstances to achieve a certain outcome [15]. The context was defined as anything that acted as either a facilitator or barrier to the implementation of the described intervention. Mechanisms were defined as how the delivered intervention produced the matched outcome [16, 22].
- The levels and types of integration of the RMIC (system, organisational, professional, and clinical level, and normative or functional) were used to arrange the items into the level on which it takes place in the transformation of the healthcare system [17]. The clinical level was interpreted as person-centredness at the individual level of citizens.
- The Framework Method was used to categorize the data into themes [23]. To enable further thematic clustering, the six elements of PHM were chosen because of the Dutch context. The six elements of PHM consist of the following:
  - ◦ The ‘accountable regional organisation’ is accountable for the Quadruple Aim and often this is a group of stakeholders that takes the form of a legal entity that can have financial arrangements with payers of health and social care.
  - ◦ The ‘cross domain business model’ is built for health and social care in the region, so that the costs and revenues resulting across different financial streams are aligned and consequences for all regional stakeholders are transparent.
  - ◦ In an ‘integrated data infrastructure’ routinely registered data in health and social care are to be connected in such a way that a regional comprehensive overview of health, costs and experiences are available.
  - ◦ ‘Co-designing workforce and community’ represents an effective structure to co-design the program and interventions with citizens and local healthcare providers ensuring direct participation and a substantial role in the final decision-making process.
  - ◦ ‘Population health data analytics’ is the use of data-driven insights to drive PHM interventions and monitor the Quadruple Aim outcomes regularly.
  - ◦ Lastly, ‘emergent implementation strategies’ are used to form a continuous process of testing and learning in the region [18].

The included articles were analysed by two researchers (AE and NM) using a standardized Excel form. To reach consensus on the classification of the data, several discussion rounds were held among the research team. During this process, the original articles were re-examined when categorisation was unclear or when there was a disagreement between the researchers. Extracting and structuring of the data was done in four steps:

- First, data were charted from the included articles as CMO combinations. These combinations were often narratives of what was done or happened (M) in which situation (C) to cause a certain effect (O) or included lessons learned of what should have been done to reach a certain outcome. This led to a list of CMO combinations retrieved from the included articles. From here on in this article, each C, M, or O is referred to as item.
- Second, with the help of the Framework Method all CMO item-combinations were grouped into themes.
- Third, all items were ordered into the RMIC. This was done for each item separately, as different levels of the RMIC played a part within a CMO combination. This also led to duplication of certain items as an outcome on one level sometimes also was a context on another level [22]. Criteria for duplication were how the items were described in the original literature or based on discussion among the research team. Consensus about the labels of the items and the assignment to the levels of the RMIC was reached through written feedback among the research team, going back to the original articles, and a final discussion within the research team.
- Finally, the thematic clustering was refined by ordering all items into the six elements of PHM. Again, some items were duplicated as they fitted in two or more PHM-elements. One researcher (AE) categorized the items into the six PHM-elements. A second researcher (VS) checked the categorisation and answered the questions. A third researcher (MB) was consulted when there was no agreement reached between the first two researchers.

Following this process led to an overview of all items that influence the implementation of PHM ordered by CMO, RMIC and the 6 elements of PHM.

Based on the preliminary results the outline for the consultation phase was discussed [20]. To complement the literature findings with experience from practice, a full study with an adjusted Delhi design was proposed. This study will be reported on separately.

## Results

### Literature search results

**Fig. 1:**
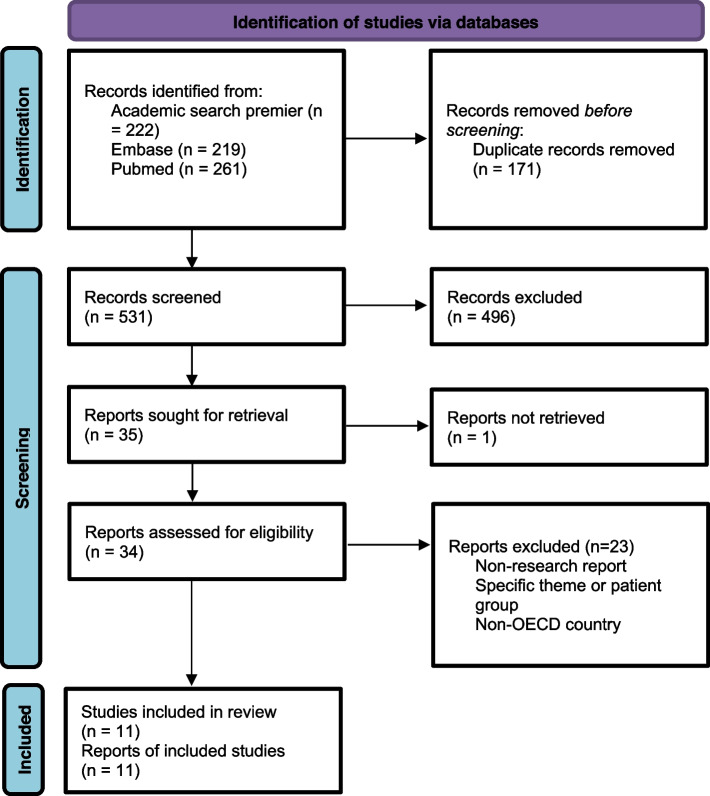
The identification of studies reported by PRISMA standards [24]

The literature search generated 531 articles from the three databases. Based on the title, 272 were selected to read the abstract. After reading the abstract, the full text was retrieved from 35 articles for in-depth screening. Despite reaching out to the author and searching by a librarian, one article was not available in full text. The screening process resulted in ten original scientific articles that described the implementation of population health management within multisector initiatives. After further discussion amongst the authors, one more article was identified as missing and added. The exclusion criteria are included in Fig. 1.

The key characteristics of the 11 included studies are described in detail in additional file 2. Most of the described interventions were implemented in the USA, the others were from Canada, Germany, the Netherlands, Singapore, and the UK [9, 25–34]. The most used methodologies were qualitative case study designs, in which interviews, focus groups, and/or document analysis were used for data collection.

### Thematic analysis of the findings

The focus in this section is on the final results after full analysis. Content analysis of the 11 articles resulted in 192 unique items that influence the transformation of a health system. After labelling and duplication using the CMO, RMIC, and PHM concepts as described above 242 items resulted from the analysis. Additional file 3 shows all items and their structuring. The following example is a quotation out of one of the included articles and the structuring of an item that was retrieved from it**:** *“Sustaining health transformation over the long term requires many staffing and capacity supports to set overall direction and integrate purpose and actions across stakeholders”* [32].

Integrate purpose and actions across stakeholders - Accountable regional organisation (six elements of PHM) – Mechanism (CMO) – System – Normative (RMIC).

**Fig. 2:**
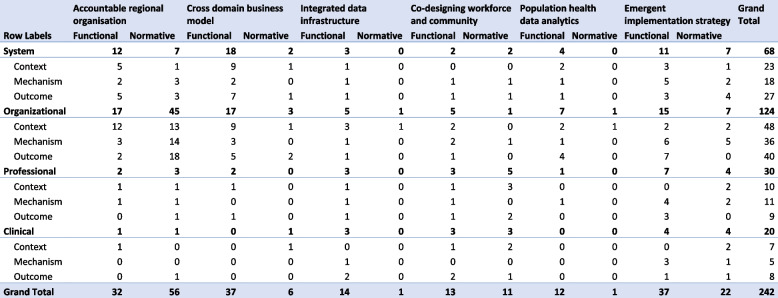
The number of items that are assigned to the different categories

Figure 2 displays a summary of the analysis by showing the distribution of the items using all three theoretical concepts. What this table demonstrates is that almost half of the reported items in the analysis, 124 items, concern the organisational level, with half of these items attributed to the PHM-element ‘accountable regional organisation’. Other high numbers, >10 items, in the table are in ‘cross domain business model’ and ‘emergent implementation strategy’, both on system and organisational level in functional integration. In contrast to that, items on the clinical level were overall underreported. In addition, only a small number of items pertained to normative integration of the PHM-elements ‘cross domain business model’, ‘integrated data infrastructure’, and ‘population health data analysis’. Thus, structuring the items according to the RMIC levels shows that all levels of the health system contribute, at least to some degree, to the implementation of PHM, but that not all items are reported on equally in the scientific literature. Whether there is an inherent bias in the literature, whether this is due to the relative importance of some items over others, or whether there are other reasons for this distribution remains unclear.

### Analysis according to the six elements of PHM and the RMIC

**Fig. 3:**
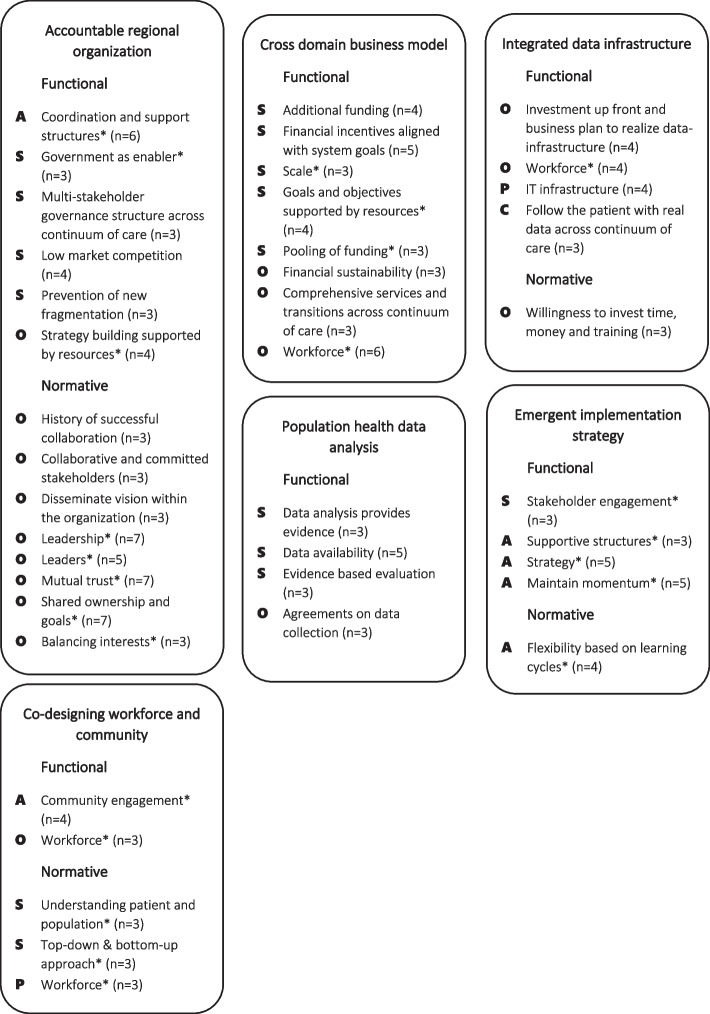
The most reported items per PHM-element. Items are only reported in this table if they were mentioned by three or more articles. The number of articles that mentioned the item is added behind each term. Terms with a * are a summary of multiple items. The items are depicted on the most relevant level of the RMIC. A= all levels, S= system, O= organisation, P= professionals, C= clinical

In Fig. 3, the results are presented differently. The table displays a summary of the most reported items per PHM-element and the classification into the RMIC, hereby presenting not only the distribution, but also showing which items were mentioned most. To give an example, ‘data availability’ in the PHM-element ‘population health data analytics’ is discussed by five out of the eleven articles taking mainly place on the system level [25, 26, 29, 33, 34]. This reveals that, although there are not as many different items mentioned within this PHM-element, some of the items were described in several articles. Therefore, this table illustrates the main focus of the initiatives found in the literature. What stands out is that there is a lot mentioned about collaboration, leadership, trust and shared ownership in ‘accountable regional organisation’, but this is not connected specifically to funding, data integration or the use of data in other PHM-elements. Another trend in this table is that the perspective of citizens and professionals is hardly visible, illustrated by the lack of P’s and C’s in the table. This table also helps breaking down the complexity of the implementation of PHM by looking at the levels of the RMIC within a PHM-element. Some items, for example ‘flexibility based on learning cycles’ in ‘emergent implementation strategy’, are mentioned on all levels [9, 28, 31, 34]. Other items, for example ‘multi-stakeholder governance structure across continuum of care’ in ‘accountable regional organisation’ and ‘financial incentives aligned with system goals’ in ‘cross domain business model’, apply mostly to one level [25, 26, 28, 30, 32–34]. This illustrates that within each PHM-element there is a focus on different aspects within different levels of the RMIC.

### Analysis according to CMO

The third concept applied to analyse the items was that of CMO. The analysis showed the interrelatedness of the items and the difficulty to classify an item as either C(ontext), M(echanism), or O(utcome). Often, the items could be all, depending on the connection to other items. This is illustrated by the following examples. Some CMO combinations are structured in the same PHM-element on the same level making it easier to order, for example in ‘accountable regional organisation’ on organisational level: within a regional organisation it is a contextual factor that ‘organisations have same motives, priorities and philosophies’ (C). This starting position supports the organisations to ‘create shared interest and aim’ (M) in order to reach a ‘position of agreement’ (O) [27, 30]. However, other items are less clear and for instance can be a C, M and O, such as ‘additional funding/monetary resources’ in ‘cross domain business model’. As context, ‘monetary resources’ (C) are a prerequisite to reach ‘investment in health system change’ (O). In another article was described that if ‘private investors within convening organisations’ (C) are present, they can provide ‘additional funding’ (O) [27, 28, 31, 32]. Another possibility that was observed, was that the CMO cuts across RMIC levels and PHM-elements. This occurs for instance in the following example: having a ‘learning environment’ (C) within the whole initiative in ‘emergent implementation strategy’, ‘secures initiatives credibility’ (O) in ‘accountable regional organisation’ by ‘supporting management and practice level by continuous improvement cycles’ (M) [9]. This displays the partial interrelatedness between levels and PHM-elements and therefore makes the complexity of implementing PHM explicit.

To summarize, by using the three models to analyse the data found in scientific literature a specific focus in the distribution of items that influence the implementation of PHM into PHM-elements, RMIC and CMO becomes apparent. The normative integration on the organisational level in ‘accountable regional organisation’ incurs the most interest both in number of items and in most frequently mentioned items. However, in the same PHM-element there are no items mentioned frequently on professional and clinical level which suggest that there is less focus on those. The lack of focus for these two levels is seen across al PHM-elements, indicating a possible gap of consideration in scientific literature at this point.

## Discussion

This study demonstrates that with the help of different theoretical concepts a further break-down can add to our understanding about what is needed on which level with what kind of mechanism to achieve a certain outcome in the implementation of PHM. Furthermore, this review shows that literature may be biased in the guidance of what matters most in the implementation of PHM. Using the six elements of PHM, the RMIC, and CMO adds to our understanding in showing this specific focus in the distribution of mentioned items. This indicates the need of further exploration of the various areas that receive little to no attention. By using the three theoretical concepts to differentiate the items that influence the implementation of PHM, a first step was taken towards a practical guide to support the implementation of PHM in practice by illustrating the depth of the complexity. Accepting that this is a complex problem in a complex adaptive system with an abundance of interrelatedness, a blueprint framework or themed list will probably not suffice [35]. As a result, this research is a first step moving away from a static to a more dynamic understanding of the implementation of PHM, and from a linear, rational process towards a learning process with continuous learning cycles.

Looking into the distribution of the items in the analysis, this review reveals several trends in the scientific literature such as the tendency for multisector initiatives to commonly report on system and organisational level activities and less on the clinical and professional levels of integration. More specifically, we encountered a focus on the normative side of the ‘accountable regional organisation’ and an absence of interest on the normative side of ‘cross domain business model’, ‘integrated data infrastructure’ and ‘population health data analysis’. These last three PHM-elements are almost exclusively reported as instruments to achieve PHM without proper normative considerations. This contrasts the perspectives of Kodner et. al. and Bengoa et. al. as they emphasize to focus also on the cultural normative aspects of these elements [36, 37]. Secondly, the importance of a combined top-down and bottom-up approach in a broader initiative as mentioned by both is also not represented in the analysis [36, 37]. To do so, more emphasis on the professional and clinical levels would be expected. This also underlines the continued lack of involvement of citizens and providers in the scientific reports of these initiatives on all levels, as has been stressed by many scholars already [11, 38]. Comparing these trends to existing frameworks, the lack of attention to these aforementioned components can be considered as gaps in design and implementation. One of the key elements of the SCIROCCO tool is ‘patient empowerment’ and the WHO frameworks starts with ‘empowering and engaging people and communities’ [12, 39]. Therefore, it seems that in multisector initiatives practice remains stuck in organising a regional governance structure on the system and organisational levels and fails to adopt a more comprehensive approach embracing the citizen’s perspective. Another explanation may be that the citizen’s perspective is present, but is not reported on in the selected articles.

A reason that the analysis is not complete, are the limitations of the chosen method. Using the scoping method made it possible to analyse and reinterpret the available literature of initiatives in Canada, the Netherlands and the USA among others. However, not all lessons learned from other health system transformation initiatives were included. This is because a lot of initiatives happening in practice are not (yet) reported in the scientific literature or are only available as conference abstracts, or are only published in reports in the local language(s), like the Belgian initiative [40]. An example of grey literature that is accessible is a report on the initiative of Gesundes Kinzigtal [41]. Additionally, articles that indeed were published were not available in the used databases, such as the initiative from the Basque Country, [42] or used other terminology to describe their initiative, such as the Vanguards programme in England [43]. These findings indicate that the multi-disciplinarity of the field of research may complicate connecting different initiatives and research to achieve knowledge synthesis. So, we urge initiators and implementors to publish all of their findings in accessible scientific literature to diminish the possibility of publication bias. Using similar terminology could be a first step to ensure reliable comparison of research findings. The encountered focus on system and organizational levels of integration in the scientific literature may be illustrative of an actual focus in implementation on these levels, or an under reporting of other levels, e.g. due to failures or encountered barriers. We challenge initiators to publish all of their findings scientifically, including the failures and barriers, to address these issues and help answer the question of whether the focus is merited or represents a bias in the literature.

This scoping review supports research in disentangling and breaking down the complexity of the PHM implementation process. While previous research only explained complexity [14] and difficulty of implementation by the interrelatedness of all items [37], this analysis attempts to disentangle the complexity with the combination of three theoretical concepts. It displays how different levels require different CMO-items depending on the six elements of PHM. However, for practice the guidance that literature can provide at the moment is insufficient on how to implement PHM. A next step could be introducing time dependency in our search of understanding the implementation of PHM. As Shaw et.al. describes, there is a connection between mechanisms and context, so that mechanisms can become a context over time or vice versa [22]. This can evolve over different levels of the RMIC or over different PHM-elements, also reflecting the dynamic character of the implementation of PHM in regional initiatives. Due to this dynamic character of the items, the phase of development may affect which items should be primarily focused on at that time. What comes first? Which items need continuous attention? And what items have a causal relation with each other? Adding this aspect of the implementation of PHM may inform practice further in their next steps of their implementation efforts. Therefore, we need accessible scientific case studies that focus on the dynamic character and the complexity of the implementation of PHM in order to get more grip on how to execute PHM in practice. For that reason, including time dependency in research can strengthen the journey in unravelling the complexity of health system transformation.

## Conclusion

This research supports initiators by adding to our understanding about the implementation of PHM and taking steps towards a dynamic tool for analysis and assessment. Using the three different theoretical concepts provided insights into the different aspects of using a PHM approach. Main lessons for practice are that, although all levels of integration are needed, there persists a specific focus per level and PHM-element. Using the separate items per level and PHM-element may guide different stakeholders or organisations in taking their next step. Next to that, initiators must be aware of the knowledge gaps in literature and take into account the normative integration and citizen’s perspective. Also, the observed interrelatedness shows that the overall implementation of PHM probably requires a collaborative effort and interconnection across levels and disciplines in order to reach health system transformation. Using the CMO concept as exercise in the local context could provide insight into the sequence and correlation of existing items. Case analysis of multisector initiatives using the three theoretical concepts can provide more insights into this complexity and sequence and guide others in the how of implementing PHM.

### Supplementary Information

**Additional file 1. **Search string.

**Additional file 2. **Key characteristics of the included studies.

**Additional file 3. **All items and their structuring according to CMO, the RMIC and the six elements of PHM.

## Data Availability

All data generated or analysed during this study are included in this published article and its supplementary information files.

## Abbreviations

CMO: Context, Mechanism, Outcome
PHM: Population Health Management
RMIC: Rainbow Model of Integrated Care
