# Supplementary material for: How to successfully implement population health management: a scoping review
Source: BMC Health Serv Res. 2023 Aug 25;23:910. doi: 10.1186/s12913-023-09915-5 (PMC10464069; doi:10.1186/s12913-023-09915-5)
Supplement: Supplementary file 1 — Additional file 1. Search string. [file 12913_2023_9915_MOESM1_ESM.docx]

# Additional file 1; search string

This file displayes the search string as was used in Pubmed.

(((("Population Health Management"[mesh] OR population health management[tw] OR population management[ti]) AND ("Triple aim"[tw] OR ("transforming health"[tw] OR "transforming healthcare"[tw] OR ((transform[ti] OR transforming[ti]) AND (health[ti] OR healthcare[ti] OR care[ti]) AND (system[ti] OR systems[ti]))) OR "Delivery of Health Care, Integrated"[Mesh] OR integrated care[tw] OR "Accountable Care Organizations"[Mesh] OR "accountable care"[tw] OR "accountable healthcare"[tw] OR "accountable health care"[tw] OR (sustainab*[ti] AND (health system[ti] OR health systems[ti] OR healthcare system[ti] OR healthcare systems[ti] OR "wellbeing system"[ti] OR "wellbeing systems"[ti] OR "health and wellbeing system"[ti] OR "health and wellbeing systems"[ti])) OR "Health Care Reform"[Mesh])) OR ("Triple aim"[tw] AND (("transforming health"[tw] OR "transforming healthcare"[tw] OR ((transform[ti] OR transforming[ti]) AND (health[ti] OR healthcare[ti] OR care[ti]) AND (system[ti] OR systems[ti]))) OR "Delivery of Health Care, Integrated"[Mesh] OR integrated care[tw] OR "Accountable Care Organizations"[Mesh] OR "accountable care"[tw] OR "accountable healthcare"[tw] OR "accountable health care"[tw] OR (sustainab*[ti] AND (health system[ti] OR health systems[ti] OR healthcare system[ti] OR healthcare systems[ti] OR "wellbeing system"[ti] OR "wellbeing systems"[ti] OR "health and wellbeing system"[ti] OR "health and wellbeing systems"[ti])) OR "Health Care Reform"[Mesh])) OR (("transforming health"[tw] OR "transforming healthcare"[tw] OR ((transform[ti] OR transforming[ti]) AND (health[ti] OR healthcare[ti] OR care[ti]) AND (system[ti] OR systems[ti]))) AND ("Delivery of Health Care, Integrated"[Mesh] OR integrated care[tw] OR "Accountable Care Organizations"[Mesh] OR "accountable care"[tw] OR "accountable healthcare"[tw] OR "accountable health care"[tw] OR (sustainab*[ti] AND (health system[ti] OR health systems[ti] OR healthcare system[ti] OR healthcare systems[ti] OR "wellbeing system"[ti] OR "wellbeing systems"[ti] OR "health and wellbeing system"[ti] OR "health and wellbeing systems"[ti])) OR "Health Care Reform"[Mesh]))) OR (("Delivery of Health Care, Integrated"[Majr] OR integrated care[ti]) AND ("Accountable Care Organizations"[Majr] OR "accountable care"[ti] OR "accountable healthcare"[ti] OR "accountable health care"[ti] OR (sustainab*[ti] AND (health system[ti] OR health systems[ti] OR healthcare system[ti] OR healthcare systems[ti] OR "wellbeing system"[ti] OR "wellbeing systems"[ti] OR "health and wellbeing system"[ti] OR "health and wellbeing systems"[ti])) OR "Health Care Reform"[majr])) AND population[tw]) OR (("Accountable Care Organizations"[mesh] OR "accountable care"[tw] OR "accountable healthcare"[tw] OR "accountable health care"[tw]) AND (sustainab*[tw] AND (health system[tw] OR health systems[tw] OR healthcare system[tw] OR healthcare systems[tw] OR "wellbeing system"[tw] OR "wellbeing systems"[tw] OR "health and wellbeing system"[tw] OR "health and wellbeing systems"[tw]))) OR ((sustainab*[ti] AND (health system[tw] OR health systems[tw] OR healthcare system[tw] OR healthcare systems[tw] OR "wellbeing system"[tw] OR "wellbeing systems"[tw] OR "health and wellbeing system"[tw] OR "health and wellbeing systems"[tw])) AND "Health Care Reform"[majr])) OR (("Population Health Management"[majr] OR population health management[ti] OR population management[ti]) OR ("Population Health"[majr:NoExp] AND improvement*[ti]) OR population health improvement*[ti] OR SCMO[tiab] OR ReThinkHealth OR ((population[ti] AND oriented[ti] AND integrated[ti] AND (care[ti] OR healthcare[ti])) OR ("population oriented"[tw] AND ("integrated care"[tw] OR "integrated healthcare"[tw] OR "integrated health care"[tw])) OR ("Delivery of Health Care, Integrated"[Mesh] AND "population oriented"[tw])) OR (Manchester[tiab] AND Devolution[tiab]) OR ((Gesundes[tiab] OR healthy[tiab]) AND Kinzigtal[tiab]) OR ("Basque experience"[tw] OR ("Basque"[tiab] AND population health[tw])))) NOT (diabet*[ti] OR neoplas*[ti] OR cancer*[ti] OR tumor*[ti] OR tumour[ti] OR malignan*[ti] OR syndrome*[ti] OR renal[ti] OR kidney*[ti] OR liver[ti] OR breast[ti] OR alcohol*[ti] OR nonacohol*[ti] OR smoking[ti] OR smoke*[ti] OR lung[ti] OR pulmonar*[ti] OR pneumon*[ti] OR asthma*[ti] OR COPD[ti] OR rheuma*[ti] OR arthrit*[ti] OR neuropath*[ti] OR neurolog*[ti] OR colorectal*[ti] OR cardiovasc*[ti] OR cardiometabol*[ti] OR bowel[ti] OR intestin*[ti] OR inflammat*[ti] OR allerg*[ti] OR metaboli*[ti] OR opioid*[ti] OR abus*[ti] OR anticoagulat*[ti] OR hypertens*[ti] OR vitamin*[ti] OR oral[ti] OR injury[ti] OR injuries[ti] OR wound[ti] OR wounds[ti] OR healing*[ti] OR brain[ti] OR rhino*[ti] OR urolog*[ti] OR orthoped*[ti] OR orthopaed*[ti] OR animal*[ti] OR dog[ti] OR dogs[ti] OR deer[ti] OR deers[ti] OR cat[ti] OR cats[ti] OR mouse[ti] OR mice[ti] OR rat[ti] OR rats[ti] OR rodent*[ti] OR pig[ti] OR pigs[ti] OR wildlife*[ti] OR bacteria[majr] OR "Biological Science Disciplines"[Majr] OR "Eukaryota"[Majr] OR genetic*[ti] OR genet*[so] OR natural disaster*[ti] OR (animals[mesh] NOT humans[mesh]))
