# Supplementary material for: How to successfully implement population health management: a scoping review
Source: BMC Health Serv Res. 2023 Aug 25;23:910. doi: 10.1186/s12913-023-09915-5 (PMC10464069; doi:10.1186/s12913-023-09915-5)
Supplement: Supplementary file 2 — Additional file 2. Key characteristics of the included studies. [file 12913_2023_9915_MOESM2_ESM.docx]

# Additional file 2; key characteristics of the included studies

In this table, the key characteristics of the included studies are described.

| **First author and reference** | **Type of research** | **Type of PHM project** | **Progress of transition** | **Country** |
| --- | --- | --- | --- | --- |
| Caldararo (2017) [25] | Qualitative data from structured interviews with 10 leaders or executives. | Leaders and executives with varying titles and experience and are currently working in organizations that are at the forefront of population health management (8 not-for-profit health care delivery systems, 1 population health institute and 1 health services research center). | Not applicable | USA |
| Farmanova (2016) [26] | A summative evaluation (site reporting documents and 15 semi-structured telephone interviews with team members) of the 9 teams of the Canadian cohort. | Structured support for TA design, implementation, evaluation and sustainability was addressed in a collaborative program of webinars and action periods. The nine Canadian teams were coached to undertake and test small-scale improvements before attempting to scale. | All sites build small-scale interventions to later on expand to a larger population. | Canada |
| Grembowski (2018) [27] | A qualitative comparative case study (reviewed documents, conducted internet searches of public information, interviewed key informants annually in 2014–2016, cross-validated factual and narrative interpretation, and performed content analyses). | Following an arduous, 6-year policy-making process, Vermont is the first state implementing a unified, statewide all-payer integrated delivery system with value-based payment, along with aligned medical and social service reforms, for almost all residents and providers in a state. 10 conditions that increased readiness of Vermont were described. | Vermont’s lessons learned just before the launch of its statewide integrated delivery system with all-payer, population-based payments. | Vermont, USA |
| Hester (2018) [28] | Not described. | Lessons learned from Vermonts experience as a laboratory for health reform, population health initiative. | The population whose care is now under the Blueprint umbrella has significantly lower total annual per capita spending ($482 or 7.0 percent) compared to a matched control group. These savings have been driven by lower inpatient use and pharmacy costs. | Vermont, USA |
| Matthews (2017) [29] | Case study. Not described. | Mayo Clinic - Mayo Model of Community Care: Mayo Clinic is a nonprofit, integrated group practice wherein providers from every medical specialty work together to meet the needs of the patient, with support from common systems and a core value that emphasizes that the needs of the patient come first. Approximately 3800 physicians and scientists and 50,900 allied health staff work at Mayo Clinic, which has academic centers in Rochester, Minnesota, Jacksonville, Florida, and Phoenix and Scottsdale, Arizona. | The mayo clinic enterprise solution was built on three phases; Basic foundation – transitional volume to value – transformation. | USA |
| Ong (2018) [30] | Qualitative study of 35 semi-structured interviews with stakeholders throughout Singapore’s health system, including representatives from all six RHS clusters, government agencies, and the private and voluntary welfare sectors, but mainly clinicians. | Transformation from 6 regions to 3 in Singapore. The RHS was described as an opportunity to holistically care for a person across the care continuum, address social determinants of health, develop new models of care, and work with social and community partners. | Implementation of re-clustering the RHS | Singapore |
| Rutledge (2019) [31] | Key informant interviews, focus groups, document review, and difference in-difference analyses using data from Medicaid claims and an all-payer claims database. Interviewees included state officials, payers and purchasers, health system and provider organizations, provider associations, advocacy groups, consumers, and consumer advocates. | Medicaid accountable care organizations in Maine, Massachusetts, Minnesota and Vermont. The purpose of this study was to identify achievements and challenges with ACO implementation and to assess the impact of Medicaid ACOs on health care utilization, quality, and expenditures in three states. | Four states demonstrated that adoption of ACOs for Medicaid beneficiaries was both possible and, for three states, associated with some improvements in care. | USA |
| Siegel (2018) [32] | Mixed-method: Nomination of partnerships, initial screening, assessing development by conducting interviews, site visits | Evaluation of regional multisector partnerships and their maturity across the USA. We designed this study to critically assess the characteristics of partnerships that have a reputation for being highly developed, with the goal of informing better fitting approaches to support their success. | In reality, the vast majority of partnerships were in early phases of development, with only a small number poised to enter or already in later phases. (overly ambitious expectations) | USA |
| Steenkamer (2020) [33] | An international, explorative comparative case study. Semi-structured interviews with 20 stakeholders from different sectors from 4 programs. | 4 different PHM programs; Greater Manchester, Vancouver Health City, Gen-H Cincinnati, Gesundes Kinzigtal. | Different stages of implementation and stakeholders from different sectors involved. | USA, UK, Canada, Germany |
| Suter (2017) [34] | An established framework and a Delphi survey with integration experts were used to identify relevant measurement domains. | no specific project, experts in the field and literature. | Not applicable | (Canada) |
| van Vooren (2020) [35] | A realist evaluation approach with three interview rounds. | In 2013, nine of these initiatives were assigned by the Dutch Ministry of Health, Welfare and Sports as ‘pioneer sites’ with the aim of developing better healthcare with lower costs. | The pioneer sites were not (yet) able to fully develop towards transition to a health and wellbeing system. | The Netherlands |
