# Supplementary material for: How to successfully implement population health management: a scoping review
Source: BMC Health Serv Res. 2023 Aug 25;23:910. doi: 10.1186/s12913-023-09915-5 (PMC10464069; doi:10.1186/s12913-023-09915-5)
Supplement: Supplementary file 3 — Additional file 3. All items and their structuring according to CMO, the RMIC and the six elements of PHM. [file 12913_2023_9915_MOESM3_ESM.docx]

# Additional file 3; all items and their structuring according to CMO, the RMIC and the six elements of PHM

This table lists all the items that where retrieved from the included articles and their structuring.

| **Term** | **PHM-element** | **CMO** | **Rainbow level** | **Normative/**  **Functional** | **Article** |
| --- | --- | --- | --- | --- | --- |
| Proactive towards federal and private demands | Accountable regional organisation | Context | Organizational | Functional | Caldararo |
| Distributed change management | Accountable regional organisation | Mechanism | Organizational | Normative | Caldararo |
| Adjustment to the era of care management | Accountable regional organisation | Mechanism | Organizational | Functional | Caldararo |
| Uniform strategy across the care continuum | Accountable regional organisation | Outcome | Organizational | Functional | Caldararo |
| Collaborative leadership | Accountable regional organisation | Context | Organizational | Normative | Caldararo |
| Align inpatient and outpatient strategy, vision and execution style | Accountable regional organisation | Mechanism | Organizational | Normative | Caldararo |
| Mutual understanding of norm, values, roles and creation of trust | Accountable regional organisation | Mechanism | Organizational | Normative | Caldararo, Grembrowski |
| Working towards a common goal | Accountable regional organisation | Outcome | Organizational | Normative | Caldararo |
| Leaders that encourage and support change at the highest level of the organization | Accountable regional organisation | Context | Organizational | Normative | Caldararo |
| Time, resources and teamwork are necessary to build a cohesive strategy | Accountable regional organisation | Context | Organizational | Functional | Caldararo, Grembrowski |
| Make the organization known as a leader in the area | Accountable regional organisation | Mechanism | Organizational | Normative | Caldararo |
| Be known as truly caring for the population | Accountable regional organisation | Outcome | Organizational | Normative | Caldararo |
| Leadership support for teams | Accountable regional organisation | Mechanism | Professional | Normative | Farmanova |
| Engaging leadership protects dedicated staff time | Accountable regional organisation | Mechanism | Organizational | Normative | Farmanova |
| Forging external partnerships | Accountable regional organisation | Mechanism | System | Normative | Farmanova |
| Macro-integrator to pull resources together on organizational level | Accountable regional organisation | Context | Organizational | Normative | Farmanova, Siegel |
| Managing competing partner priorities and values, role clarity and conflict resolution | Accountable regional organisation | Mechanism | Organizational | Normative | Farmanova |
| Formal (leadership) structures and processes | Accountable regional organisation | Context | Organizational | Functional | Farmanova, Ong |
| Coordination across levels of all sectors implicating health | Accountable regional organisation | Context | Clinical | Functional | Farmanova |
| Coordination across levels of all sectors implicating health | Accountable regional organisation | Context | Organizational | Functional | Farmanova |
| Coordination across levels of all sectors implicating health | Accountable regional organisation | Context | System | Functional | Farmanova |
| New model supports retention of leaders | Accountable regional organisation | Context | Organizational | Functional | Grembrowski |
| Committed champions guides deliberations toward decisions | Accountable regional organisation | Mechanism | Organizational | Normative | Grembrowski, Van Vooren |
| Shared willingness to intervene for the common good | Accountable regional organisation | Outcome | Organizational | Normative | Grembrowski |
| Overcome setbacks and uncertainties | Accountable regional organisation | Outcome | Organizational | Normative | Grembrowski |
| Coalition of the willing | Accountable regional organisation | Outcome | Organizational | Normative | Grembrowski |
| Position of agreement | Accountable regional organisation | Outcome | Organizational | Normative | Grembrowski, Ong |
| Create shared interest and aim | Accountable regional organisation | Mechanism | Organizational | Normative | Grembrowski, Ong |
| Law and regulations to promote and support PHM | Accountable regional organisation | Context | System | Functional | Grembrowski |
| Support of legal system | Accountable regional organisation | Context | Organizational | Functional | Grembrowski |
| Support of policy system | Accountable regional organisation | Context | Organizational | Functional | Grembrowski, Van Vooren |
| Equal relationships among leaders | Accountable regional organisation | Context | Organizational | Normative | Ong |
| Organizations having same motives, priorities and philosophies | Accountable regional organisation | Context | Organizational | Normative | Ong |
| Strong organizational culture | Accountable regional organisation | Outcome | Organizational | Normative | Ong |
| Strong organizational culture | Accountable regional organisation | Context | Professional | Normative | Ong |
| Shared sense of importance, urgency and commitment to deliver good care to patients and populations | Accountable regional organisation | Outcome | Organizational | Normative | Ong, Steenkamer |
| Prior provider experience with new models of care | Accountable regional organisation | Context | Organizational | Normative | Rutledge |
| Pilot testing an ACO model | Accountable regional organisation | Mechanism | System | Functional | Rutledge |
| Understanding PHM rationale and trust in shared vision | Accountable regional organisation | Context | Organizational | Normative | Steenkamer |
| Commitment to the partnership | Accountable regional organisation | Outcome | Organizational | Normative | Steenkamer, Van Vooren |
| Shared ownership for achieving the initiatives goals | Accountable regional organisation | Outcome | Organizational | Normative | Steenkamer |
| Secure initiatives credibility | Accountable regional organisation | Outcome | System | Normative | Steenkamer |
| Visionairy leader from an organization not percieved as threat and supported by funders | Accountable regional organisation | Context | System | Normative | Van Vooren |
| Balancing organizational vs PHM initiatives interest | Accountable regional organisation | Mechanism | Organizational | Normative | Van Vooren |
| Address motivations of organizations that play a role in commitment | Accountable regional organisation | Mechanism | Organizational | Normative | Van Vooren |
| Share both successes and risks | Accountable regional organisation | Outcome | Organizational | Normative | Van Vooren |
| Insight in each others responsibilities | Accountable regional organisation | Outcome | Organizational | Normative | Van Vooren |
| Define preconditions for accountability | Accountable regional organisation | Mechanism | Organizational | Normative | Van Vooren |
| Supportive policy development | Accountable regional organisation | Outcome | System | Normative | Van Vooren |
| Ensure regional agreements are underpinned by political support | Accountable regional organisation | Mechanism | System | Normative | Van Vooren |
| Leaders that take risks | Accountable regional organisation | Context | Organizational | Normative | Van Vooren, Siegel |
| Build on existing care coordination structures | Accountable regional organisation | Mechanism | Organizational | Functional | Hester |
| Trust and certainty among stakeholders | Accountable regional organisation | Outcome | Organizational | Normative | Hester, Siegel |
| State government as enabler | Accountable regional organisation | Context | Organizational | Functional | Hester |
| Supported top-level executives | Accountable regional organisation | Outcome | Organizational | Normative | Matthews |
| PHM advisory group to top-level executives | Accountable regional organisation | Context | Organizational | Functional | Matthews |
| Strong leaders in key positions in governance support strucure | Accountable regional organisation | Context | System | Functional | Matthews |
| Sound governance support system | Accountable regional organisation | Outcome | System | Functional | Matthews |
| Leverage current governance structures | Accountable regional organisation | Mechanism | Organizational | Functional | Matthews |
| Operations leadership for each clinical program | Accountable regional organisation | Context | Professional | Functional | Matthews |
| Support an enterprise approach and keep PHM initiatives moving forward | Accountable regional organisation | Outcome | Organizational | Normative | Matthews |
| Attainment of goals and objectives are supported by funding and human resource allocation | Accountable regional organisation | Outcome | Organizational | Functional | Matthews, Suter |
| Integrate purpose and actions across stakeholders | Accountable regional organisation | Mechanism | System | Normative | Siegel |
| Coordinate: spot connections, reinforce a common narrative, drive a comprehensive action agenda | Accountable regional organisation | Mechanism | Organizational | Normative | Siegel |
| Involve all key players in the region with high-level leaders | Accountable regional organisation | Mechanism | System | Functional | Siegel |
| Promoting active conflict management | Accountable regional organisation | Mechanism | Organizational | Normative | Siegel |
| Partnerships with important structure, authority and resources | Accountable regional organisation | Outcome | System | Functional | Siegel |
| Health reform efforts by state | Accountable regional organisation | Context | System | Functional | Siegel |
| Trusted relationships | Accountable regional organisation | Outcome | Clinical | Normative | Siegel |
| Trusted relationships | Accountable regional organisation | Outcome | Organizational | Normative | Siegel |
| Trusted relationships | Accountable regional organisation | Outcome | Professional | Normative | Siegel |
| Trusted relationships | Accountable regional organisation | Outcome | System | Normative | Siegel |
| Similarities of goals, mission and vision | Accountable regional organisation | Context | Organizational | Normative | Suter |
| Shared protocols based on evidence | Accountable regional organisation | Context | Organizational | Functional | Suter |
| Organizational goals and objectives aligned across sectors | Accountable regional organisation | Outcome | Organizational | Normative | Suter |
| Organizational culture congruent with vision | Accountable regional organisation | Mechanism | Organizational | Normative | Suter |
| Committed leadership recognizes the importance of learning | Accountable regional organisation | Context | Organizational | Normative | Suter |
| Strategic alliances government, public and external stakeholders | Accountable regional organisation | Outcome | System | Functional | Suter |
| Multi-stakeholder governance structure for continuum of care | Accountable regional organisation | Context | Organizational | Functional | Farmanova, Siegel, Suter |
| Multi-stakeholder governance structure for continuum of care | Accountable regional organisation | Outcome | System | Functional | Farmanova, Siegel, Suter |
| Coordination across levels of all sectors implicating health | Accountable regional organisation | Mechanism | Professional | Functional | Farmanova, Ong |
| Collaborative and committed stakeholders | Accountable regional organisation | Outcome | Organizational | Normative | Grembrowski, Ong, Van Vooren |
| History of successful collaboration | Accountable regional organisation | Context | Organizational | Normative | Grembrowski, Hester, Siegel |
| Competence of leaders; expertise, charisma, experience, not reluctant to take on risks | Accountable regional organisation | Context | Organizational | Normative | Caldararo, Van Vooren, Siegel |
| Prevention of new fragmentation | Accountable regional organisation | Outcome | System | Functional | Ong, Steenkamer, Siegel |
| Leaders that disseminate the vision to all levels of the organization | Accountable regional organisation | Context | Organizational | Normative | Caldararo, Farmanova, Suter |
| Low market competition | Accountable regional organisation | Context | Organizational | Functional | Caldararo, Grembrowski, Ong, Siegel |
| Low market competition | Accountable regional organisation | Context | System | Functional | Caldararo, Grembrowski, Ong, Siegel |
| Involved physicians in clinical initiatives and protocol development | Co-designing workforce and community | Context | Organizational | Functional | Caldararo |
| Understanding population needs | Co-designing workforce and community | Outcome | System | Functional | Farmanova, Van Vooren |
| Engagement of provider | Co-designing workforce and community | Context | Professional | Functional | Farmanova |
| Community engagement | Co-designing workforce and community | Context | Clinical | Functional | Farmanova, Suter |
| Community engagement | Co-designing workforce and community | Mechanism | Organizational | Functional | Grembrowski |
| Community engagement | Co-designing workforce and community | Mechanism | System | Functional | Grembrowski |
| Top-down and bottom-up approach | Co-designing workforce and community | Mechanism | System | Normative | Grembrowski, Van Vooren |
| Increased patient satisfaction | Co-designing workforce and community | Outcome | Clinical | Normative | Ong |
| Enabled and empowered workforce | Co-designing workforce and community | Outcome | Professional | Normative | Ong, Matthews |
| Workforce work with new paradigm | Co-designing workforce and community | Outcome | Professional | Normative | Rutledge, Matthews |
| Top-down and bottom-up approach | Co-designing workforce and community | Mechanism | Organizational | Normative | Steenkamer |
| Co-creative interaction to come with the best model for change | Co-designing workforce and community | Mechanism | Organizational | Functional | Steenkamer |
| Professionals and community awareness of required attitudes and behaviors | Co-designing workforce and community | Outcome | System | Normative | Steenkamer |
| Community engagement | Co-designing workforce and community | Context | Organizational | Functional | Van Vooren |
| Leader with decision making power, trust of peers and credibility around clinical and technical integration | Co-designing workforce and community | Context | Professional | Normative | Matthews |
| Physician executive leadership | Co-designing workforce and community | Context | Professional | Normative | Matthews |
| Ensure knowledge sharing possibilities between different working groups | Co-designing workforce and community | Mechanism | Professional | Functional | Siegel |
| Understanding of the patient needs and the way they move within the system | Co-designing workforce and community | Context | Clinical | Normative | Suter |
| Patient and/or family involvement in care planning | Co-designing workforce and community | Outcome | Clinical | Functional | Suter |
| Workforce perspective; professional autonomy, clear roles/responsibilities, equality among workforce | Co-designing workforce and community | Context | Clinical | Normative | Suter |
| Effective leadership among workforce | Co-designing workforce and community | Context | Professional | Normative | Suter |
| Physician integration within care teams and across sectors | Co-designing workforce and community | Outcome | Clinical | Functional | Suter |
| Physician integration within care teams and across sectors | Co-designing workforce and community | Outcome | Organizational | Functional | Suter |
| Physician integration within care teams and across sectors | Co-designing workforce and community | Outcome | Professional | Functional | Suter |
| Limit financial downside of risks | Cross domain business model | Mechanism | Organizational | Functional | Caldararo |
| Stakeholders open to share data | Cross domain business model | Context | Organizational | Normative | Caldararo |
| Financial capacity to take risks | Cross domain business model | Context | Organizational | Functional | Caldararo, Van Vooren |
| Micro-integrators on clinical level to coordinate services | Cross domain business model | Context | Clinical | Normative | Farmanova |
| Working in multi-disciplinairy teams | Cross domain business model | Outcome | Organizational | Normative | Farmanova |
| Accountability and alignment of team members’ expertise with the portfolio to capitalize on existing capacity | Cross domain business model | Outcome | Organizational | Normative | Farmanova |
| Build a businesscase based on population needs | Cross domain business model | Mechanism | Organizational | Functional | Farmanova |
| Small area | Cross domain business model | Context | System | Functional | Grembrowski, Siegel |
| Design and implementation of payment reform strategies | Cross domain business model | Outcome | System | Functional | Grembrowski |
| Law and regulations to promote and support PHM | Cross domain business model | Context | System | Functional | Grembrowski |
| Align services with population needs | Cross domain business model | Outcome | Organizational | Functional | Grembrowski |
| Skilled workforce that can adapt to changing system | Cross domain business model | Context | Professional | Functional | Grembrowski |
| Sufficient human resources | Cross domain business model | Context | Organizational | Functional | Ong |
| Investment in manpower and development and training programs | Cross domain business model | Context | Organizational | Functional | Ong |
| Central manpower pool that can be deployed to partner institutions where needed | Cross domain business model | Mechanism | Organizational | Functional | Ong |
| Active support for education and training of future workforce | Cross domain business model | Context | System | Functional | Ong |
| Incentives for patients seeking care to end up with the right provider | Cross domain business model | Outcome | System | Functional | Ong |
| Significant coordination across payers | Cross domain business model | Outcome | System | Normative | Rutledge |
| Align operational aspects of different payment programs | Cross domain business model | Mechanism | System | Functional | Rutledge |
| Investment in health system change | Cross domain business model | Outcome | System | Functional | Rutledge |
| Common vision on payment reform | Cross domain business model | Context | System | Normative | Steenkamer, Van Vooren |
| Private investors within convening organizations | Cross domain business model | Context | System | Functional | Steenkamer |
| Pooling of budgets across different municipal departments | Cross domain business model | Context | System | Functional | Steenkamer |
| Mitigating the effects of political changes and elections | Cross domain business model | Context | System | Functional | Steenkamer |
| Economy of scale | Cross domain business model | Context | System | Functional | Steenkamer |
| Corporate presence in the region | Cross domain business model | Context | Organizational | Functional | Steenkamer |
| Availability of resources (time, money, personnel) | Cross domain business model | Context | System | Functional | Steenkamer, Van Vooren |
| Staffing and capacity alterations | Cross domain business model | Context | Organizational | Functional | Van Vooren, Siegel |
| Skilled workforce that can adapt to changing system | Cross domain business model | Context | Organizational | Functional | Van Vooren |
| Attainment of goals and objectives are supported by funding and human resource allocation | Cross domain business model | Outcome | Organizational | Functional | Matthews, Suter |
| Cover regional governance financial needs | Cross domain business model | Outcome | System | Functional | Siegel |
| System-wide financial planning | Cross domain business model | Outcome | System | Functional | Siegel |
| Standardised care delivery through interprofessional teams | Cross domain business model | Outcome | Professional | Functional | Suter |
| Management allows pooling of funds across services | Cross domain business model | Context | Organizational | Functional | Suter |
| Integrated service funding across sectors | Cross domain business model | Context | Organizational | Functional | Suter |
| Additional funding/monetary resources | Cross domain business model | Context | System | Functional | Grembrowski, Rutledge, Hester, Siegel |
| Additional funding/monetary resources | Cross domain business model | Mechanism | System | Functional | Grembrowski, Rutledge, Hester, Siegel |
| Additional funding/monetary resources | Cross domain business model | Outcome | System | Functional | Grembrowski, Rutledge, Hester, Siegel |
| Comprehensive services and transitions across the continuum of care; between sectors and providers, also supporting services as education and social services | Cross domain business model | Outcome | Organizational | Functional | Grembrowski, Rutledge, Suter |
| Financial incentives aligned with system goals | Cross domain business model | Context | Organizational | Functional | Caldararo, Ong, Van Vooren, Hester |
| Financial incentives aligned with system goals | Cross domain business model | Outcome | Organizational | Functional | Caldararo, Ong, Van Vooren, Hester |
| Financial incentives aligned with system goals | Cross domain business model | Outcome | System | Functional | Caldararo, Ong, Van Vooren, Hester,Suter |
| Financial sustainability | Cross domain business model | Outcome | Organizational | Functional | Steenkamer, Hester, Siegel |
| PHM and value-based healthcare strategies | Emergent implementation strategy | Outcome | Organizational | Functional | Caldararo |
| PHM and value-based healthcare strategies | Emergent implementation strategy | Outcome | System | Functional | Caldararo |
| Create and communicate a focused strategy with well-defined and measurable goals | Emergent implementation strategy | Mechanism | Organizational | Normative | Caldararo |
| Support to prioritize and focus on core responsibilities | Emergent implementation strategy | Outcome | Professional | Functional | Farmanova |
| Structured and evidence based approach to support scale-up and sustainability | Emergent implementation strategy | Outcome | Organizational | Functional | Farmanova |
| Level playing field | Emergent implementation strategy | Outcome | Clinical | Functional | Farmanova |
| Level playing field | emergent implementation strategy | Outcome | Organizational | Functional | Farmanova |
| Level playing field | emergent implementation strategy | Outcome | Professional | Functional | Farmanova |
| Level playing field | emergent implementation strategy | Outcome | System | Functional | Farmanova |
| Engagement of policy maker | emergent implementation strategy | Context | System | Functional | Farmanova |
| Capacity building to understand, adapt and apply triple aim in practice | Emergent implementation strategy | Mechanism | Clinical | Functional | Farmanova |
| Capacity building to understand, adapt and apply triple aim in practice | Emergent implementation strategy | Mechanism | Organizational | Functional | Farmanova |
| Capacity building to understand, adapt and apply triple aim in practice | Emergent implementation strategy | Mechanism | Professional | Functional | Farmanova |
| Capacity building to understand, adapt and apply triple aim in practice | Emergent implementation strategy | Mechanism | System | Functional | Farmanova |
| Engagement of other stakeholders | emergent implementation strategy | Context | Organizational | Functional | Farmanova, Siegel |
| Sustained momentum towards system reform | Emergent implementation strategy | Outcome | System | Normative | Grembrowski, Van Vooren |
| Statewide involvement on the perspective on reform | Emergent implementation strategy | Outcome | System | Normative | Grembrowski |
| Prioritize local needs | emergent implementation strategy | Mechanism | Organizational | Normative | Grembrowski |
| Align services with population needs | Emergent implementation strategy | Outcome | Organizational | Functional | Grembrowski |
| Availability of multidisciplinairy teams | Emergent implementation strategy | Context | Professional | Normative | Grembrowski, Ong |
| Increase HR efficiency | Emergent implementation strategy | Outcome | Organizational | Functional | Ong |
| Care optimization | Emergent implementation strategy | Mechanism | Clinical | Functional | Ong |
| Care optimization | Emergent implementation strategy | Mechanism | Professional | Functional | Ong |
| Ability to change service delivery | Emergent implementation strategy | Mechanism | Clinical | Normative | Ong |
| Ability to change service delivery | Emergent implementation strategy | Mechanism | Professional | Normative | Ong |
| Change way of thinking towards patient-centricity | Emergent implementation strategy | Outcome | Clinical | Normative | Ong, Suter |
| Change way of thinking towards patient-centricity | Emergent implementation strategy | Mechanism | Professional | Normative | Ong, Suter |
| Decreasing duplication, increasing efficiency in patient care process | Emergent implementation strategy | Outcome | Organizational | Functional | Ong |
| Decreasing duplication, increasing efficiency in patient care process | Emergent implementation strategy | Mechanism | Professional | Functional | Ong |
| Change way of thinking towards patient-centricity | Emergent implementation strategy | Mechanism | Organizational | Normative | Ong |
| A culture of person-centered, holistic care | Emergent implementation strategy | Context | Clinical | Normative | Ong |
| States decision to pursue a new health model based on prior provider experience | Emergent implementation strategy | Context | System | Functional | Rutledge |
| Incentives to work with the new paradigm | Emergent implementation strategy | Context | Organizational | Normative | Rutledge |
| Incentives to work with the new paradigm | Emergent implementation strategy | Mechanism | System | Normative | Rutledge |
| Programmatic revisions, quality improvements, provider networks/support | Emergent implementation strategy | Outcome | System | Functional | Rutledge |
| Programmatic flexibility | Emergent implementation strategy | Mechanism | Organizational | Normative | Rutledge |
| Support management and practice level by continuous improvement cycle | Emergent implementation strategy | Mechanism | Organizational | Functional | Steenkamer |
| Support management and practice level by continuous improvement cycle | Emergent implementation strategy | Mechanism | System | Functional | Steenkamer |
| Learning environment | Emergent implementation strategy | Context | Clinical | Normative | Steenkamer |
| Learning environment | emergent implementation strategy | Context | Organizational | Normative | Steenkamer |
| Learning environment | emergent implementation strategy | Context | Professional | Normative | Steenkamer |
| Learning environment | emergent implementation strategy | Context | System | Normative | Steenkamer |
| Develop supportive structures and processes for training, measurement, monitoring and information flows | Emergent implementation strategy | Mechanism | Organizational | Functional | Steenkamer |
| Develop supportive structures and processes for training, measurement, monitoring and information flows | Emergent implementation strategy | Mechanism | System | Functional | Steenkamer |
| Invest in public campaign | Emergent implementation strategy | Mechanism | System | Functional | Steenkamer |
| Ensure a learning cycle | emergent implementation strategy | Mechanism | Organizational | Normative | Van Vooren |
| Spreading the network and share experience | Emergent implementation strategy | Mechanism | System | Normative | Hester |
| Learning network | Emergent implementation strategy | Context | System | Functional | Hester |
| Prove the systems value and use the learning experience | Emergent implementation strategy | Outcome | System | Normative | Hester |
| Implement a first wave of interventions promptly | Emergent implementation strategy | Mechanism | System | Functional | Hester |
| Decision making and communication between levels | Emergent implementation strategy | Mechanism | Clinical | Functional | Matthews |
| Decision making and communication between levels | Emergent implementation strategy | Mechanism | Organizational | Functional | Matthews |
| Decision making and communication between levels | Emergent implementation strategy | Mechanism | Professional | Functional | Matthews |
| Meet scope and objectives | Emergent implementation strategy | Outcome | Organizational | Functional | Matthews |
| Meet scope and objectives | Emergent implementation strategy | Outcome | Professional | Functional | Matthews |
| Keep implementation on target | Emergent implementation strategy | Mechanism | Organizational | Functional | Matthews |
| Broad strategy that encompass both healthcare delivery reform and upstream efforts beyond individual social services | Emergent implementation strategy | Outcome | System | Normative | Siegel |
| Addressing nonclinical factors that influence health | Emergent implementation strategy | Context | Organizational | Functional | Siegel |
| Addressing nonclinical factors that influence health | Emergent implementation strategy | Mechanism | Organizational | Functional | Siegel |
| Law and regulations to promote and support PHM | Integrated data infrastructure | Context | System | Functional | Caldararo, Grembrowski |
| IT infrastructure | Integrated data infrastructure | Outcome | Organizational | Functional | Caldararo, Ong |
| Prevention of new fragmentation | Integrated data infrastructure | Context | Organizational | Functional | Ong |
| Information sharing between and among workforce | Integrated data infrastructure | Mechanism | Professional | Functional | Ong |
| Drive evidence based and best practices decision making | Integrated data infrastructure | Outcome | Professional | Functional | Ong, Matthews |
| Skilled workforce, e.g. professional data manager | Integrated data infrastructure | Context | Organizational | Functional | Rutledge, Van Vooren |
| Developing a data and knowledge infrastructure | Integrated data infrastructure | Mechanism | System | Functional | Van Vooren |
| Comprehensive clinical picture of the patient | Integrated data infrastructure | Outcome | Clinical | Functional | Matthews |
| System-wide information system across sectors | Integrated data infrastructure | Outcome | System | Functional | Suter |
| Incentives workforce to fill in standard data collection | Integrated data infrastructure | Context | Organizational | Functional | Suter |
| Shared information accessible to patients | Integrated data infrastructure | Outcome | Clinical | Functional | Suter |
| IT infrastructure | Integrated data infrastructure | Context | Professional | Functional | Caldararo, Ong, Rutledge, Matthews |
| Willingness to invest time, money and training | Integrated data infrastructure | Context | Organizational | Normative | Caldararo, Rutledge, Suter |
| Follow the patient with real time data across continuum of care | Integrated data infrastructure | Mechanism | Clinical | Functional | Caldararo, Rutledge, Matthews |
| Investment up front and business plan to realize data infrastructure | Integrated data infrastructure | Mechanism | Organizational | Functional | Caldararo, Rutledge, Hester, Suter |
| Stakeholders open to share data | Population health data analytics | Context | Organizational | Normative | Caldararo |
| Produce concise and useful datasets | Population health data analytics | Outcome | Organizational | Functional | Caldararo |
| Geographically or discretely defined population | Population health data analytics | Context | System | Functional | Farmanova |
| Data availability | Population health data analytics | Context | Organizational | Functional | Grembrowski |
| Develop better understanding of health beneficiaries and their needs | Population health data analytics | Outcome | Organizational | Functional | Grembrowski, Ong |
| Patient stratification | Population health data analytics | Mechanism | Professional | Functional | Ong |
| Data used for service planning | Population health data analytics | Outcome | Organizational | Functional | Suter |
| Agreements on data collection | Population health data analytics | Context | Organizational | Functional | Farmanova, Matthews, Suter |
| Evidence based evaluation | Population health data analytics | Outcome | System | Functional | Farmanova, Siegel, Suter |
| Data analysis provides evidence | Population health data analytics | Mechanism | Organizational | Functional | Caldararo, Farmanova, Grembrowski |
| Data analysis provides evidence | Population health data analytics | Mechanism | System | Functional | Caldararo, Farmanova, Grembrowski |
| Data availability | Population health data analytics | Outcome | Organizational | Functional | Caldararo, Farmanova, Van Vooren, Matthews, Suter |
| Data availability | Population health data analytics | Context | System | Functional | Caldararo, Farmanova, Van Vooren, Matthews, Suter |
